# Association Between Left Renal Vein Entrapment and Varicocele Recurrence: A Cohort Study in 3042 Patients

**DOI:** 10.1038/s41598-018-28887-9

**Published:** 2018-07-12

**Authors:** Sen Li, Qian Liu, Jin Wang, Xueqin Pang, Youpeng Zhang, Yongbiao Cheng, Yao Fu, Jialun Guo, Yong Tang, Hanqing Zeng, Yali Yang, Zhaohui Zhu

**Affiliations:** 1 Department of Urology, Union Hospital, Tongji Medical College, Huazhong University of Science and Technology, Wuhan, 430022 China; 2 Department of Nosocomial Infection Management, Union Hospital, Tongji Medical College, Huazhong University of Science and Technology, Wuhan, 430022 China; 3 Department of Ultrasound, Union Hospital, Tongji Medical College, Huazhong University of Science and Technology, Wuhan, 430022 China

## Abstract

The recurrence rates after varicocelectomy vary from 0.9% to 32.2%, especially for patients with the left renal vein entrapment (LRVE). This study aims to study the association between LRVE and varicocele recurrence, and to find the risk factors of LRVE. With the design of a cohort study, we included 3042 varicocele patients who would undergo modified inguinal microscope-assisted varicocelectomy (MHMV). 858 (28.21%) patients with LRVE were as the study group, and 2184 (71.79%) patients without LRVE were as the control group. Compared with the control group, BMI was lower (p < 0.001) in study group. Totally, 18 patients had recurrence after surgery, so the recurrence rate was 0.59%. Seventeen patients (1.98%) in study group and 1 patients (0.05%) in control group had recurrence, and significant statistical difference was found between the two groups (p < 0.001). The risk ratio of LRVE for varicocele recurrence is 43.27. In conclusion, the recurrence rate of our MHMV is the lowest (0.59%). There is association between LRVE and varicocele recurrence, and varicocele patients with LRVE have higher probability of recurrence rate after varicocelectomy. BMI could be a risk factor of LRVE. Thus, for varicocele patients, especially those with lower BMI, attentions should be payed to LRVE.

## Introduction

Varicocele is a common abnormality of the testis characterized by excessive dilatation of the pampiniform venous plexus of the spermatic cord^1^, with the following andrological implications: failure of ipsilateral testicular growth and development, symptoms of pain and discomfort, male infertility^2^. The incidence of varicocele in male adults is approximately 15%, with a prevalence of 24–41% of men with infertility^3,4^. It is known that 85% of the patients had the varicocele on the left side^4^. Left renal vein entrapment (LRVE), defined as compression of left renal vein (LRV) between the aorta and the superior mesenteric artery (SMA), is common in varicocele patients^5,6^. For varicocele repair, a simple ligation of the left spermatic vein is an effective approach^4^. However, recurrence rates after surgical repair vary from 0.9% to 32.2%, especially for patients with LRVE^3,7^.

From 2008 to 2013, about 3000 varicocele patients underwent modified inguinal microsurgical varicocelectomy by our medical group, and we found 20 patients had recurrence. Then we found a research reported all patients with LRVE and only 20.1% of patients without LRVE had varicocele recurrence, and concluded the presence of LRVE resulted in a significantly higher varicocele recurrence rate^3^. We performed Color Doppler ultrasonography for the 20 patients, and found that 18 patients had LRVE and only 2 had no LRVE. Thus, we hypothesized that LRVE was associated with varicocele recurrence. To verify our hypothesis, we included 520 patients with varicocele from July 2013 to March 2015, and found that LRVE may be an important influence factor of recurrence in our previous study^8^. However, the sample size of the study was not large, especially for the study group. Now, we included more varicocele patients to make a more reliable and credible evaluation on the association between LRVE and varicocele recurrence, and to research the risk factors of LRVE.

## Methods

### Patient selection

From July 2013 to June 2017, we enrolled the patients who were diagnosed with clinical palpable unilateral or bilateral varicocele and underwent varicocelectomy by our medical group. According to European Association of Urology (EAU) Guideline^9^, clinical palpable varicocele has been classified into Grade I (palpable during Valsava manoeuvre, but not otherwise), Grade II (palpable at rest, but not visible) and Grade III (visible and palpable at rest).

According to EAU Guideline and Guideline for Diagnosis and Treatment of Urological Disease in China (2014), the Indications of varicocelectomy were: (1) infertility because of low semen quality; (2) scrotal pain; (3) persistent prostatitis; (4) no symptoms, but varicocele in Grade 3 was found in medical examination, and asked for varicocelectomy; (5) testicular atrophy in adolescent. When the patients had two or more indications, we chose the most important one for description.

### Study design and surgical procedures

We employed a cohort study to compare the varicocele recurrence between the varicocele patients with LRVE and those without LRVE.

The varicocele patients with LRVE were as study group, while those without LRVE were as control group. The diagnosis of varicocele is based on physical examination and ultrasound evaluation. Two radiologists with more than 10 years of experience performed the ultrasound examination on shift work. We provided same training on Color Doppler ultrasonography of LRV for the two radiologists before this study. Then, we conducted a preliminary investigation in which two radiologists performed ultrasonography of LRV on the same 100 patients, and interobserver agreement was calculated using kappa statistics (kappa values: <0 indicates poor agreement, 0.01 to 0.20 indicates slight agreement, 0.21 to 0.40 indicates fair agreement, 0.41 to 0.60 indicates moderate agreement, 0.61 to 0.80 indicates substantial agreement, and >0.80 indicate nearly perfect agreement)^10^. The kappa value was found to be 0.712, which indicates substantial agreement. Sonography of LRV was performed with patients in a supine position. The peak velocity ratio between the aortomesenteric portion (AMP, the portion of the LRV between the aorta and superior mesenteric artery) and hilar portion of the LRV was calculated, and a hilar portion peak velocity greater than 5-fold the AMP peak velocity was considered diagnostic of LRVE^11,12^. All patients of two groups underwent color Doppler ultrasonography to observe the LRVE before operation, then accepted a modified inguinal microsurgical varicocelectomy. All the surgery was conducted by one doctor. The surgical procedures were shown as follows (video):

1. The incision location was marked (A. surface projection of external inguinal ring, B. a 2-cm marking line at the direction of iliac crest with 1 cm away from the external inguinal ring (① at the 2/5 outside of the vertical distance between ipsilateral anterior superior iliac spine and median line of abdomen, ② the upper margin of incision, ③ the lower margin of incision, ④ the midpoint of the incision). Local anesthetic solution was used (20 ml of 2% lidocaine combined with 20 ml normal saline and 0.1 ml of 0.1% adrenaline).
2. A combination of local anesthesia and IV sedation was used for the patients who were either overweight (BMI ≥ 30), very nervous, or requested IV sedation. Dexmedetomidine hydrochloride injection (4ug/ml) was infused slowly with the dose of 1ug/kg, and the infusion time was more than 10 min.
3. Conventional disinfection and drape surgical towels were applied with the patient placed in supine position. The needle was injected at site ① and stopped when it passed through the abdominal external oblique aponeurosis with an obviously falling feeling. Subsequently, 9 ml of local anesthetic were injected to block the iliohypogastric nerve. The needle was injected at site ② and stopped as the same as site ①, and 9 ml of local anesthetic were injected, infiltrated layer by layer through the abdominal external oblique aponeurosis, the subcutaneous tissue and the ilioinguinal nerve. Cutaneous and subcutaneous infiltration anesthesia was performed at site ③, and 6 ml of local anesthetic were injected, infiltrated layer by layer to block subcutaneous tissue incision. Cutaneous and subcutaneous infiltration anesthesia was also performed at site ④, with 3 ml of local anesthetic injected, infiltrated layer by layer to block the skin and subcutaneous tissue of the incision.
4. The skin and subcutaneous tissue were incised, exposing the external oblique aponeurosis, and a longitudinal incision was made. Blunt dissection of the cremaster muscle was performed and spermatic cord below the muscle was identified, facilitating the placement of the spermatic cord to the skin incision.
5. The fascia of the spermatic cord was incised, and 1–2 larger spermatic veins were raised, to expose the remaining spermatic cord blood vessels and surrounding fat tissue which were raised at the same time, visualizing the obvious boundaries with the vas deferens vascular system.
6. Blunt dissection at the junction of the vas deferens vascular system was conducted, and the two layers were separated with a piece of rubber sheet.
7. The upper spermatic cord tissue was then placed under a microscope with a resolution increase of 8-fold.
8. The testicular artery and lymph vessels were carefully isolated. Vascular pulsation and blood flashing were observed or micro ultrasonic Doppler was used to confirm and protect the testicular artery. All the internal spermatic veins were ligated using Surgical Silk 5–0 and incised. Thick veins with no artery or lymph-vessel around could be ligated together.
9. Finally, a check was performed for obvious leakage and bleeding, sutured the fascia of the spermatic cord and the muscle of the testis, sutured each layer, and glued the incision.

### Assessment

We recorded the age, nationality, body mass index (BMI), varicocele side, varicocele grade, and indications of varicocelectomy of every patient. Three months after the operation, spermatic vein ultrasound was taken to know whether varicocele recurred. All patients received follow-up of 6 months after operation to observe the scrotal edema, testicular volume change and varicocele recurrence. Patients lost in the follow-up will be excluded.

### Statistical analysis

Statistical analysis was performed using SPSS version 16 software (SPSS Inc, Chicago, IL). Independent t-test test was used for the comparison of continuous variables, such as age and BMI. Chi-square test was used for the comparison of categorical variables, such as nationality, varicocele side, varicocele grade, and indications of varicocelectomy of every patient. P ≤ 0.05 was considered statistically significant. The rate of recurrence in the two groups was determined, and a risk ratio (RR) was used to describe the difference.

### Ethical consideration

Informed consent was obtained from all participants and confidentiality was ensured. The study was approved by the ethics committee of Tongji Medical College, Huazhong University of Science and Technology. Our methods were performed in accordance with the European Association of Urology (EAU) Guidelines, and performing varicocelectomy for patients with persistent prostatitis and clinical palpable varicocele conformed to the Guideline for Diagnosis and Treatment of Urological Disease in China.

### Data availability

The datasets generated during and/or analyzed during the current study are available from the corresponding author on reasonable request.

## Results

Totally, 3298 patients underwent varicocelectomy by our medical group, and 163 patients were lost in the follow-up of six months. Thus, 3042 (92.24%) patients were included. Among them, 858 (28.21%) patients were with LRVE, and the other 2184 (71.79%) were without LRVE.

As Table 1 shows, there was no statistical difference in age, nationality, varicocele side, varicocele grade, indications of varicocelectomy, and peak velocity at hilar portion of LRV between the two groups. But we found statistical difference in BMI between two groups, and BMI of patients with LRVE was lower than that of those without LRVE (p < 0.001). Patients with LRVE had higher peak velocity at AMP than those without LRVE (p < 0.001), and peak velocity ratios of patients with LRVE was higher than those without LRVE (p < 0.001).

**Table 1:** Basic information of the varicocele patients.

	Patients with LRVE	Patients without LRVE	X^2^/t	P-value
**Total, n(%)**	858 (28.21)	2184 (71.79)		
Age, yr				
Mean ± SD	29.71 ± 8.62	30.92 ± 8.31	1.805	0.071
Range	11–62	10–66		
Nationality				
China	854	2176	0.156	0.693
Others	4	8		
Varicocele, n(%)				
Left	448 (52.21)	1144 (52.38)		
Right	12 (1.40)	52 (2.38)	3.007	0.222
Bilateral	398 (46.39)	988 (45.24)		
Varicocele grade, n(%)				
I	524 (61.07)	1292 (59.24)		
II	146 (17.02)	388 (17.77)	0.943	0.624
III	188 (21.91)	504 (23.08)		
BMI, kg/m^2^				
Mean ± SD	22.12 ± 2.92	24.61 ± 3.22	3.292	<0.001*
Range	15.98–27.84	17.43–30.73		
Cat. BMI, n(%)				
15-	104 (12.12)	180 (8.24)		
20-	612 (71.33)	1366 (62.55)	56.415	<0.001*
25-	142 (16.55)	634 (29.03)		
30–35	0	4 (0.18)		
Indications, n(%)				
Infertility	582 (67.83)	1516 (69.41)		
Scrotal pain	160 (18.65)	358 (16.39)	4.510	0.341
Persistent prostatitis	90 (10.49)	258 (11.81)		
With no symptoms	18 (2.10)	32 (1.47)		
Testicular atrophy in adolescent	8 (0.93)	20 (0.92)		
**Peak velocity at AMP, cm/s**	105.52 ± 25.48	38.59 ± 21.44	3.291	<0.001*
**Peak velocity at hilar portion, cm/s**	17.30 ± 3.64	18.12 ± 6.15	1.635	0.108
**Peak velocity ratios**	6.58 ± 1.44	2.09 ± 1.00	3.296	<0.001*

As shown in Table 2, among the 858 patients with LRVE, 15 had left-sided recurrence, 1 patient had right-sided recurrence, 1 had bilateral recurrence, and the remaining 841 patients had no recurrence. Thus, the recurrence rate of the study group was 1.98% (17/858). While among the 2184 patients with no LRVE, only 1 had left-sided recurrence, and the remaining 2183 patients had no recurrence. The recurrence rate of the control group was 0.05% (1/2184). Thus, the RR of LRVE for varicocele recurrence is 43.27. There was statistical difference in recurrence between the two groups (p < 0.001). The recurrence rate of all the varicocele patients was 0.59% (18/3042). In addition, 11 patients (1.28%) with LRVE and 30 patients (1.37%) without LRVE had hydrocele after varicocelectomy, and no statistical difference was found (p = 0.843).

**Table 2:** Varicocele recurrence and hydrocele of the patients after varicocelectomy.

	Patients with LRVE, n(%)	Patients without LRVE, n(%)	X^2^	P-value
**Varicocele recurrence**	17 (1.98)	1 (0.05)	39.234	<0.001*
Left-sided recurrence	15 (1.75)	1 (0.05)		
Right-sided recurrence	1 (0.12)	0		
Bilateral recurrence	1 (0.12)	0		
**No varicocele recurrence**	841 (98.14)	2183 (99.95)		
**Hydrocele**	11 (1.28)	30 (1.37)	0.039	0.843

## Discussion

There are anatomical differences between left and right renal venous drainage. The route of left spermatic vein is longer than the right side, and the left spermatic vein was vertically injected into the LRV which cause higher pressure in LRV. These factors lead to increased pressure transmission to the left scrotum vein and the occurrence of varicose veins^13^. Furthermore, LRV is compressed when flowing into the inferior vena cava through the angle between the abdominal aorta and SMA, affecting the vein reflux, accompanied by the renal vein dilation^14,15^. As our results showed, the left-side varicocele was much more than right-side. The incidence of LRVE in the normal male adults was 2.3%, but the incidence of LRVE in varicocele patients was 28.21% in our study. It is similar with a previous study which reported that the proportion of patients with varicocele that had sonographic findings indicative of the nutcracker phenomenon was more than 30%^11^.

When LRVE causes a series of symptoms such as hematuria, orthostatic proteinuria, lumbago and so on, it is known as Nutcrackers syndrome (NCS)^16^. For the therapy of NCS patients combined with varicocele, there is still no consensus. Patients with Mild NCS can take active observation, as the collateral circulation increases with age and the increase of fat connective tissue around superior mesenteric artery can relieve LRVE. But the infertility caused by varicocele has not been resolved^17^. For some patients with persistent or repeated clinical symptoms, conservative treatment is ineffective, and surgical treatment or interventional therapy should be taken. But these surgical procedures have the shortcomings of serous trauma and many complications^18^. In recent years, LRV stentimplantation is carried out as minimally invasive surgery with the advantages of small trauma, quick recovery and good short-term effect, but its long-term efficacy needs to be followed up^19^.

The spermatic vein ligation is the most commonly used surgical approach for varicocele, with high incidence of recurrence up to 25%, and 68% of recurrence happened because the miss ligation of testicular venous branches. It has been reported that microscopic spermatic vein ligation is more complete, and can retain the spermatic cord and lymphatic vessels, whose effect is better, recurrence and complications rate are lower^20^. The main reasons for varicocele recurrence are: (1) Spermatic vein ligation is incomplete; (2) There are extensive ramus anastomoticus among the internal spermatic vein, vas deferens vein, external spermatic vein, abdominal superficial vein; (3) Obstructive lesions of the inferior vena cava, common iliac vein, internal iliac and external iliac vein^21^. Our study focused on the relationship between LRVE and varicocele postoperative recurrence. Secondary varicocele was excluded for all the enrolled cases. The data showed that the recurrence rate was 1.98% in the varicocele patients with LRVE, much higher than 0.05% of the control group. The difference was statistically significant (p < 0.001). We may conclude that LRVE will increase the risk of postoperative recurrence in varicocele patients. Therefore, we recommend all varicocele patients receive LRV color Doppler ultrasound examination before surgery. For the thin and tall varicocele patients with LRVE, we suggest them to increase weight appropriately^22^. For the varicocele patients with LRVE, microscopic spermatic vein ligation of varicocele treatment is still a good choice due to the low recurrence rate of 1.98%.

Our results showed that the recurrence rate of our modified inguinal microsurgical varicocelectomy under local anaesthesia was 0.59%. According the previous researches, the recurrence rates of microsurgical subinguinal varicocelectomy^23^, nonmagnified subinguinal varicocelectomy^24^, and laparoscopic varicocelectomy^25^ was 3.6%, 11.3%, and 17%, respectively. Compared with these, the recurrence rates our modified inguinal microsurgical varicocelectomy is the lowest. We found the ratio of hydrocele was 1.28% and 1.37% for patients with LRVE and those without LRVE, respectively, which is similar with a previous study^24^. No statistical difference on hydrocele rate was found between the two groups (p = 0.843), which indicates LRVE may not be related to hydrocele after varicocelectomy.

For the treatment of varicocele patients combined with LRVE in the absence of significant hematuria or proteinuria, the effect of simple microscopic spermatic vein ligation is not clear so far. One study reported that if the varicocele patients with NCS received simple ligation of spermatic vein, the degree of LRVE elevated, LRV blood flow increased, hematuria or proteinuria aggravated, and varicocele easily relapsed. LRV displacement, stent implantation and spermatic vein bypass surgery must be used to solve the problem completely^26^.

Our evolution of varicocelectomy has many advantages. Firstly, we use local anesthesia which could not only shorten the operation time, but also reduce the patients’ pain of fasting and catheterization, and they can return to normal activities immediately after surgery. Secondly, we can see the 2–8 spermatic veins in inguinal region. The diameter of the vein is larger, so it is easy to be separated. The number of internal spermatic vein is obviously less than that of the external inguinal ring. Thirdly, the ilioinguinal/iliohypogastric nerve block was performed to ease the discomfort when we divided and ligated the spermatic cord. The genitofemoral nerve block was performed to reduce the pain of incision and scrotum. Furthermore, we conducted intradermal suture and tissue glue incision for small and slight scar. Finally, there is no leakage, and the recurrence rate was very low.

The results showed that statistical difference exists in BMI between two groups, and BMI of patients with LRVE (22.12 ± 2.92) was lower than that of those without LRVE (24.61 ± 3.22) (p < 0.001). In a previous study conducted in Turkey from 2006 to 2010, S. Gorur *et al*. found that the BMI score was found as a determinant for varicocele recurrence and BMI score lower than 25 kg/m^2^ significantly increases the recurrence rate after varicocele operation^27^, which is consistent with our study.

Our research has some strengths and limitations. First, based on the hypothesis from about 3000 patients, we got a large sample of 3042 patients in the cohort study. Second, all the participants were patients admitted to the same medical group of our hospital, so it is limited in only one medical group of one hospital, and it may lead to Berkson’s bias. Even though, the same surgeons and the same surgical approach contributed to the stable and reliable results. Although it is probably by accident that only one patient with LRVE had right-sided recurrence and anther one had bilateral recurrence among all the patients, further research could be conducted on this problem.

## Conclusions

Patients with LRVE account for 28.21% of all varicocele patients. The recurrence rate of our modified inguinal microsurgical varicocelectomy is the lowest (0.59%). There is association between LRVE and varicocele recurrence, and varicocele patients with LRVE have higher probability of recurrence rate after varicocelectomy. BMI could be a risk factor of LRVE. Thus, for varicocele patients, especially those with lower BMI, attentions should be payed to LRVE.

## Electronic supplementary material

The surgical procedures of varicocelectomy
